# A qPCR identification scheme to detect the most common causative agents of actinomycetoma in Africa

**DOI:** 10.1371/journal.pntd.0014710

**Published:** 2026-09-08

**Authors:** Andrew K. Watson, Sahar Bakhiet, Emmanuel E. Siddig, Carole Pab Minlekib, Rihab Mohammed, Willemien Zandijk, Mickey Konings, Ahmed H. Fahal, Maguette Faye, Katarzyna Mickiewicz, Fatehmeh Mazraati Tajabadi, Doudou Sow, Jeffrey Errington, Wendy W. J. van de Sande

**Affiliations:** 1 Centre for Bacterial Cell Biology, Biosciences Institute, Newcastle University, Newcastle upon Tyne, United Kingdom; 2 ErasmusMC, Department of Medical Microbiology and Infectious Diseases, Rotterdam, The Netherlands; 3 Mycetoma Research Centre, University of Khartoum, Khartoum, Sudan; 4 The Institute of Endemic Diseases, University of Khartoum, Khartoum, Sudan; 5 Faculty of Medical laboratory Sciences, University of Khartoum, Khartoum, Sudan; 6 Centre de Recherche sur les maladies Tropicales Négligées, UFR Sciences de la Santé, Université Gaston Berger, Saint-Louis, Senegal; 7 Department of medical microbiology and parasitology, faculty of Medical Laboratory sciences, University of Dongola, Dongola, Sudan; 8 Sydney Institute for Infectious Diseases, Faculty of Medicine and Health, University of Sydney, Sydney, NSW, Australia; Yale University, UNITED STATES OF AMERICA

## Abstract

Mycetoma is a neglected tropical disease characterized by mutilating tumorous lesions in the subcutaneous tissue. The causative agents are found embedded in granules called grains. Mycetoma is either caused by bacteria (actinomycetoma) or fungi (eumycetoma). To initiate the appropriate treatment, it is important to identify the causative agent rapidly and molecular identification for eumycetoma revolutionized the time to identification. For actinomycetoma this was not possible yet. Here we developed a multiplex qPCR identification scheme for the most common causative agents of actinomycetoma in Africa. Whole genome sequencing was used to identify species-specific gene families for *Actinomadura madurae, Actinomadura pelletieri, Streptomyces somaliensis* and *Streptomyces sudanensis*. qPCR primers and probes were developed on these species and validated against DNA isolated from mycetoma strains and grains. Each probe was unique with no cross-reactivity with other tested species. The limit of detection ranged from 0.000013 to 0.00067 ng bacterial DNA. When the qPCRs were validated against 28 grain samples, all fungal grains remained negative and 11 out of 12 *Actinomadura* grains were correctly identified. This resulted in a sensitivity of 85.7% for the *A. pelletieri* probe and a specificity of 100%. For the *A. madurae* probe, a sensitivity and specificity of 100% was obtained. The actinomycetoma qPCR developed in this study can be used to identify the most common causative agents of actinomycetoma in Africa.

## Author summary

Mycetoma is a devastating disease with large tumorous lesions. It can be caused by fungi and bacteria. Most laboratories use either histology and/or culturing to identify the causative agents, but these procedures can take up to six weeks till a positive identification is obtained. Recently we demonstrated that qPCR can be used directly on mycetoma grains and can be used to discriminate actinomycetoma from eumycetoma within 2 hours. Furthermore, for actinomycetoma identification to the genus level can be achieved with this qPCR. However, if a species identification was needed there were no qPCR assays available to achieve this. Therefore, in this project we aimed to develop qPCR methods to identify the most common causative agents of actinomycetoma in Africa. Instead of using classical barcode genes, we searched the genomes of *Actinomadura madurae, Actinomadura pelletieri, Streptomyces somaliensis* and *Streptomyces sudanensis* to find conserved unique genes for each lineage. qPCRs were designed on these genes and validated against a large cohort of fungal and bacterial mycetoma causative agents. No cross reaction was observed. Next, we also validated these qPCRs against the mycetoma grains. In these clinical samples a sensitivity of 85.7% and a specificity of 100% was achieved. The next step is to validate this qPCR identification scheme throughout different mycetoma endemic regions in Africa and beyond.

## Introduction

In 2016, mycetoma was recognized as a Neglected Tropical Disease (NTD) by the World Health Organization (WHO) [1]. The disease is characterized by large tumorous lesions in the subcutaneous tissue, and most often these lesions are found on the feet [1]. Especially when the lesions become large, they hinder the patients in their daily activities [1]. So far, 90 different micro-organisms have been described in the literature as mycetoma causative agents, all of which organize themselves in tissue in protective structures called grains [2]. In 42% of all mycetoma cases, the causative agent is a fungus (eumycetoma), while in 51% of cases the causative agent is a bacterium (actinomycetoma). In 7% of cases this was not specified [2]. Eumycetoma is treated with a combination of antifungal treatment, 400 mg/day itraconazole, and surgery [3]. Under the conditions of a clinical trial this resulted in cure for 80% of patients [3]. In case series this resulted in corrected cure rates ranging from 9.1% to 67.6% [4]. Actinomycetoma is most commonly treated with five-week antibiotic cycles in which 42 mg/kg/day trimethoprim-sulfamethoxazole is given for five weeks and 15 mg/kg/day amikacin only in the first three weeks of this cycle [5]. In 90% of patients complete cure is reached within three 5-week cycles [5,6]. However, differences are noted between the different causative agents [7–9]. In general actinomycetoma caused by *Actinomadura* spp is more difficult to treat than actinomycetoma caused by *Nocardia* spp [7], despite similar *in vitro* susceptibility patterns [9]. In Africa, actinomycetoma is often treated with a combination of trimethoprim-sulfamethoxazole and amoxicillin-clavulanic acid. For amoxicillin-clavulanic acid, species specific differences in susceptibility were noted. All tested *S. somaliensis* and *S. sudanensis* isolates were susceptible towards amoxicillin/clavulanic acid while only 55% of the tested *A. madurae* and 61.5% of the tested *N. brasiliensis* isolates were susceptible towards this drug [9].

To initiate the appropriate treatment, it is important to identify the causative agent rapidly. Therefore the WHO has established a Target Product Profile (TPP) emphasizing the need for an accessible *in vitro* point-of-care test capable of identifying mycetoma causative agents to the species level [10]. In Africa, diagnosis is established by a combination of clinical examination, histopathology and culture [11]. In some centers ultrasound is also used [12]. With histopathology it is possible to discriminate actinomycetoma and eumycetoma, but identification to the species level is not possible [12]. An average of 8.5 days was needed before the histopathology results were obtained [12]. Furthermore, with histopathology false negative results were reported in 146 out of 750 *M. mycetomatis* cases, 18 out of 71 *A. madurae* cases, 4 out of 16 *A. pelletieri* cases and 9 out of 142 *S. somaliensis* cases [13]. With culture it is possible to identify to the species level, however due to the slow growth rates of most causative agents an average of 21.2 days was needed before this identification was made for fungal causative agents [12]. For bacterial causative agents the average time to identification was not reported. The fungal time-to-identification is still above the minimal requirements as set in the TPP.

**Fig 1:**
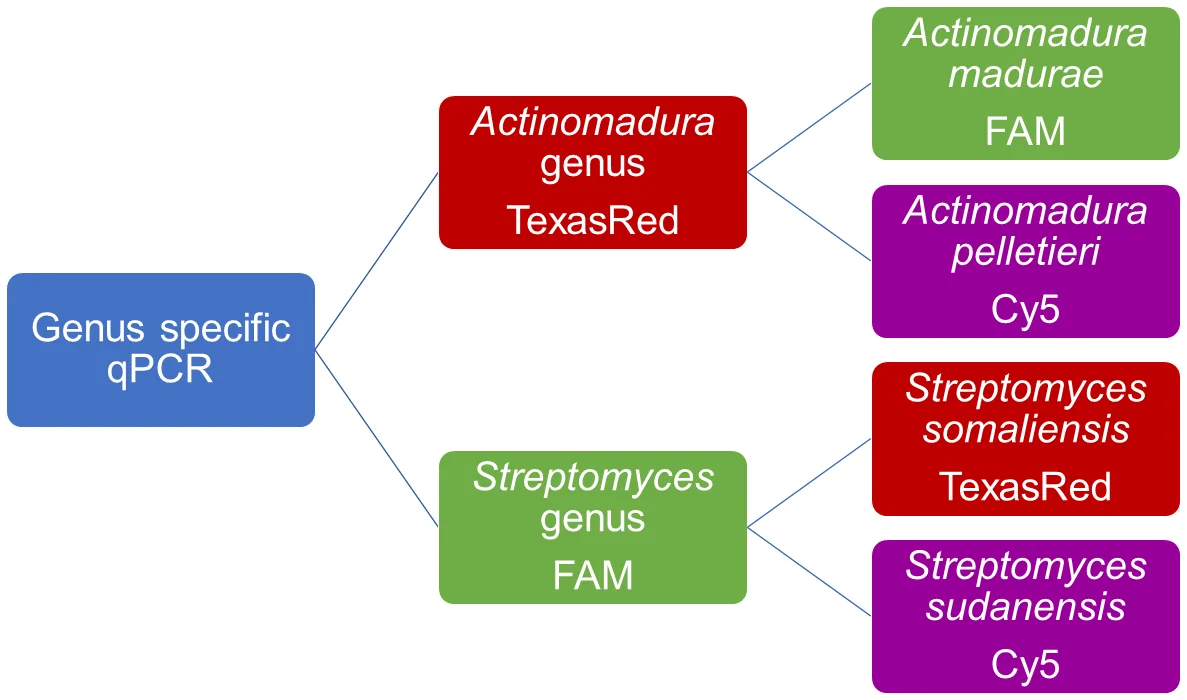
qPCR identification scheme to identify the most common causative agents of actinomycetoma in Africa. In the proposed qPCR scheme first the recently developed genus specific qPCR will be performed [14]. In case the *Actinomadura* genus qPCR is positive, this will be followed by a qPCR targeting *A. madurae* or *A. pelletieri.* In case the *Streptomyces* genus qPCR is positive, this will be followed by a qPCR targeting *A. somaliensis* or *S. sudanensis.*

In order to improve this, we recently developed a qPCR which could differentiate eumycetoma from actinomycetoma in less than 4h [14]. Furthermore, in the same qPCR the genus for the actinomycetoma causative agents was determined [14]. In this study we aimed to complement this identification scheme to be able to identify the actinomycetoma causative agents not only to the genus level but also to the species level (Fig 1). However, the actinomycetoma causative agents are not evenly distributed over the world. In Latin-America, *Nocardia brasiliensis* is by far the most common causative agent [2]. In contrast, in Africa, *N. brasiliensis* is hardly encountered and *Streptomyces somaliensis, Streptomyces sudanensis, Actinomadura madurae* and *Actinomadura pelletieri* are most common [2,15]. Due to the many misidentifications encountered, we started by building a collection of actinomycetoma causative agents from patient isolates and reference collections and sequenced their complete genomes. Since in our consortium we only saw patients from Senegal and Sudan, we developed species specific qPCRs for *A. madurae, A. pelletieri, S. somaliensis* and *S. sudanensis,* the most common causative agents of actinomycetoma in Africa (Fig 1). The genome data obtained from these isolates was subsequently used to identify species specific coding regions that differentiate the most common causative agents. qPCRs were developed on these coding regions and validated against a large cohort of mycetoma associated isolates and finally, in Senegal, directly on clinical material.

## Materials and methods

### Ethics statement

This study was performed in line with the principles of the Declaration of Helsinki. Approval was granted by the National Ethical Committee of Senegal (Comité National d’Ethique pour la Recherce en Santé; CNERS) (No 0000063/MSAS/CNERS/SP) for the Senegalese isolates and grains and from the Soba University Hospital Ethical committee (Khartoum, Sudan) for the Sudanese isolates. Written informed consent was obtained from all individual participants included in the study.

### Bacterial and fungal strains and grains

For this study we used a collection of 28 grains, 91 bacterial and 63 fungal isolates of mycetoma causative agents. An overview of the number of included isolates is provided in Table 1, and a full overview of included isolates is provided in S1 Table. An overview of the grain samples is provided in Table 2 and S2 Table. Bacterial isolates were maintained on BBL Trypticase Soy Agar with 5% Sheep Blood (Becton Dickinson, USA) at 37°C. Fungal isolates were maintained on Sabouraud Dextrose Agar (Becton Dickinson, USA) at 37°C or 21°C (Room temperature) depending on the species.

**Table 1:** Isolates used in this study.

Species	Bacterial/Fungal	Known mycetoma causative agent#	Number of isolates	Number of samples correctly identified by species specific qPCR
*Actinocorallia weidayensis*	Bacterial	No*	1	NA
*Actinomadura cremea*	Bacterial	No	1	NA
*Actinomadura geliboluensis*	Bacterial	Yes	2	NA
*Actinomadura hallensis*	Bacterial	No	1	NA
*Actinomadura latina*	Bacterial	Yes	1	NA
*Actinomadura madurae*	Bacterial	Yes	19	19
*Actinomadura meyerae*	Bacterial	No	2	NA
*Actinomadura pelletieri*	Bacterial	Yes	13	13
*Actinomadura welshii*	Bacterial	Yes	2	NA
*Nocardia asteroides*	Bacterial	Yes	1	NA
*Nocardia brasiliensis*	Bacterial	Yes	9	NA
*Nocardia farcinica*	Bacterial	Yes	1	NA
*Nocardia mexicana*	Bacterial	Yes	1	NA
*Nocardia vulneris*	Bacterial		2	NA
*Sphaerosporangium* spp	Bacterial	No	1	NA
*Streptomyces albidoflavus*	Bacterial	No*	2	NA
*Streptomyces albogriseolus*	Bacterial	No*	1	NA
*Streptomyces chrestomyceticus*	Bacterial	No	1	NA
*Streptomyces griseus*	Bacterial	No	1	NA
*Streptomyces kordofanensis*	Bacterial	No*	1	NA
*Streptomyces radiopugnans*	Bacterial	No*	1	NA
*Streptomyces somaliensis*	Bacterial	Yes	7	7
*Streptomyces sudanensis*	Bacterial	Yes	19	19
*Streptomyces werraensis*	Bacterial	No*	1	NA
*Exophiala oligosperma*	Fungal	No*	1	NA
*Falciformispora senegalensis*	Fungal	Yes	6	NA
*Falciformispora tompkinsii*	Fungal	Yes	2	NA
*Madurella fahalii*	Fungal	Yes	3	NA
*Madurella mycetomatis*	Fungal	Yes	35	NA
*Madurella pseudomycetomatis*	Fungal	Yes	7	NA
*Madurella tropicana*	Fungal	Yes	2	NA
*Medicopsis romeroi*	Fungal	Yes	1	NA
*Nigrograna mackinnonii*	Fungal	Yes	1	NA
*Scedosporium boydii*	Fungal	Yes	1	NA
*Trematosphaeria grisea*	Fungal	Yes	4	NA

#A species was considered a known causative agent of mycetoma if it was published in at least a case report as being a mycetoma causative agent

*Although these species were not known as mycetoma causative agents, the isolates used in this study were isolated from mycetoma patients

**Table 2:** 28 clinical grain samples from Senegal used in this study.

Causative agent identified by barcode sequencing	Grain colour	Number of samples	Number of samples correctly identified by species specific qPCR at ErasmusMC	Number of samples correctly identified by species specific qPCR at endemic setting
*Actinomadura geliboluensis*	white	1	0	1
*Actinomadura madurae*	white	3	3	3
*Actinomadura pelletieri*	red	7	7	6*
*Actinomadura* spp	white	1	0	1
*Madurella fahalii*	black	1	NA	NA
*Madurella mycetomatis*	black	10	NA	NA
*Madurella pseudomycetomatis*	black	3	NA	NA
*Madurella tropicana*	black	1	NA	NA
*Medicopsis romeroi*	black	1	NA	NA

*the seventh isolate was identified to the genus level, but not to the species levelNA: not applicable

### DNA isolation

DNA was isolated from a single grain, 0.25 cm^2^ fungal colony or 3 bacterial colonies using the Fungal/Bacterial DNA MicroPrep kit (Zymo Research, USA) according to the manufacturer’s instructions, with one exception: for grains and fungal isolates the bashing beads were replaced by 10 metal beads. After isolation, the DNA concentration was determined using a NanoDrop and a Quant-iT PicoGreen dsDNA Assay Kit (Invitrogen, USA), according to the manufacturer’s instructions. Each DNA sample was diluted to a final concentration of 2.5 ng/µl. Positive control samples were prepared from 2.5 ng/µl of *A. madurae* DSM43067 DNA, 2.5 ng/µl *N. brasiliensis* ATCC19296 DNA and 2.5 ng/µl of *S. somaliensis* DSM40738 DNA. These positive controls were used in every qPCR reaction.

### Sequencing and taxonomic annotation

Sequencing and genome assembly was carried out by MicrobesNG (Birmingham, United Kingdom). Sequencing libraries were generated using SQK-RBK114.96. Sequencing was performed on a GridION (Oxford Nanopore Technologies) using an R10.4.1 flowcell, with basecalling model r1041_e82_400bps_hac_v4.2.0. Reads were randomly subsampled to 50X coverage using Rasusa (V 0.7.1) [https://doi.org/10.21105/joss.03941] and assembled using Flye (V2.9.2-b1786) [https://doi.org/10.1038/s41587-019-0072-8]. Assemblies were polished using Medaka (V 1.8.0) and the relevant model (r1041_e82_400bps_hac_v4.2.0). Genome annotations were inferred using bakta (version 1.8.1, Database v 5.0) [https://doi.org/10.1099/mgen.0.000685]. Contigs were annotated as chromosomal or plasmid derived using the MOB-suite mob-recon took, with default settings [16]. Whole genome sequences of newly sequenced isolates, combined with previously sequenced mycetoma isolates from the MRC [17] were used as queries in an initial taxonomic annotation of genomes using the Genome Taxonomy Database (GTDB) toolkit (version 2.1.1 [18]) to search against GTDB release 214 [19]) in both default mode, and using the de novo phylogeny workflow, with a number of dependencies [20–26]. Using the results from this search, genus specific datasets were established that included all new genomes sequenced in this project assigned to that genus by GTDBtk, as well as all related genomes identified by GTDBtk. For each genus specific dataset, a de novo phylogenetic tree of marker genes from the GTDB bac120 marker set was inferred using iqtree2 [27] with automatic model selection from ModelFinder [28] and the following parameters (alrt 1000; bb 1000). Additionally, % average nucleotide identity (ANI) for all pairwise comparisons of genomes in the datasets were calculated using fastANI [29]. Finally, DDH values between isolates and their closest relative in GTDBtk and the single genome were estimated using GGDC [30,31]. As previously [17] ANI > 95% (and DDH > 70% were used to delineate species boundaries. Further, S3 Table includes columns indicating the probability that isolates represent new sub-species based on DDH values [32].

*Actinomadura* remains the valid genus name for mycetoma agents *A. madurae* and *A. pelletieri* according to the Accepted Lists and LPSN [33]. However, within the GTDB the genus *Actinomadura* has been merged with several others based on their similarity at the whole genome level. This new merged genus is called *Spirillospora* in GTDB [19]. We use the GTDB taxonomic system for the purposes of interacting with their database and selecting genomes related to our mycetoma isolates. However, we have continued to use the genus name *Actinomadura* for our isolates, in line with LPSN recommendations.

### *A. welshii* genome reassembly

The publicly available long-read sequence data (BioProject PRJNA1232214) associated with the recently published genome of the newly identified causative agent of mycetoma, *A. welshii* [34], was reassembled using Flye version (parameters) [35,36]. The new assembly was used for all genome comparisons presented in this manuscript, is available in supplementary data (https://figshare.com/s/e2ce5c77aad51dd942ee) and has been submitted to NCBI as a potential update to the existing reference genome.

### Identification of species-specific gene families

Phylogenetic order specific datasets were compiled that include the highest quality genome sequence available for every species within the order Streptomycetales and Streptosporangiales (as defined in GTDB release 214 [19]), which respectively include the genera *Streptomyces* and *Actinomadura* (https://figshare.com/s/e2ce5c77aad51dd942ee)*.* The quality of genome sequences was assessed using the checkM estimated % completeness and % contamination scores provided by GTDB using the following formula: Quality = Completeness-(5*Contamination). Any genomes with a quality score <90 or without protein sequence annotations available in NCBI were excluded from analysis. Additionally, every sequenced genome available for target mycetoma agents at the time of analysis was included in these datasets. The identification of candidate target genes was carried out prior to the availability of the new genome sequences identified in this study but includes isolates from Watson et al., 2022 [17].

For Streptomycetales, the final dataset included a total of 626 reference genomes, with the addition of 10 isolate genomes from Watson et al., 2022. For Streptosporangiales the final dataset included 109 reference genomes, with the addition of 7 isolate genomes from Watson et al., 2022. An additional dataset was also assembled that sampled 695 genomes from a broad taxonomic range across the phylum Actinomycetota, with the addition of the same 17 mycetoma isolate sequences.

To select target genes, we identified families of genes that were specific to individual mycetoma agents, but that were universally conserved in all sequenced representatives of their species with a high degree of sequence similarity. First, we identified gene families that were specific to the species compared to sequenced representatives of other species within the same genus. Next, we checked whether these gene families were species-specific in a broader dataset that sampled across Actinobacteria as a whole. For each dataset, all protein sequences from all genomes were used as both query and database in all-vs-all BLAST search using diamond blastp (more-sensitive mode, minimum of 30% identity and an E. value < 1x10^-7^). Genes were clustered into gene families based on the results of this similarity search using MCL with an inflation parameter of 1.2. The resultant gene families were screened using a custom python script to identify any gene families specific to a particular mycetoma agent at the species level, first in its phylogenetic order specific dataset, and then this was cross-checked against the broader dataset encompassing a range of Actinomycetoma. Finally, we checked the distribution of these gene families in the genomes of newly sequenced isolates presented in this manuscript. Gene families that only included sequences from a specific mycetoma agent, and that were universally conserved amongst sequenced isolates from these species (including newly sequenced isolates presented in this manuscript), were carried forwards for further analysis. From initial sets of candidate species-specific gene families identified in this search, priority was given to the genes with the highest % sequenced similarity in pairwise comparisons of sequences from genomes of the target species. A final manual BLAST search against NR was used to curate the selection and ensure the distribution of selected gene families was sufficiently narrow.

### Design and validation of the qPCR

To design the primers and probes each of the selected DNA sequences was loaded into Primer3 (version 0.4.0; https://bioinfo.ut.ee/primer3-0.4.0/). Default conditions were used for the primers, meaning that the optimum primer Tm was set at 60 °C (± 3^o^C). The optimum Tm for the probe was set at 70 °C (± 3^o^C). Then, a multiple primer dimer analysis was performed using the Multiple Prime Analyzer tool available online from Thermo Fisher (https://www.thermofisher.com/nl/en/home/brands/thermo-scientific/molecular-biology/molecular-biology-learning-center/molecular-biology-resource-library/thermo-scientific-web-tools/multiple-primer-analyzer.html). To be able to use the *Actinomadura* genus and *Streptomyces* genus probe as internal positive controls the hybridizations dyes were chosen according to the schedule presented in Fig 1. Selected primers and probes are presented in Table 3.

**Table 3:** Selected primers and probes.

Multiplex nr	Target	Name	Sequence
1	*Actinomadura* and *Streptomyces* genus	Act_Hsp65_fw	5’-GTGGAGACCAAGGAGCAGAT-3’
1	*Actinomadura* and *Streptomyces* genus	Act_Hsp65_rv	5’-CTTCCTTGCCGACCTTGTC-3’
1	*Actinomadura* genus	Actinomadura_Hsp65_probe	5’ -TEXAS RED-ACCCGCAGATCGGCGAGATGAT-BHQ2–3’
1	*Streptomyces* genus	Streptomyces_Hsp65_probe	5’-6FAM-CCGCCTCCATCTCCGCC-EDQ-3’
2	*Actinomadura madurae*	Am_7737_fw	5’-ATCAAGCTCCCGGTCCAG-3’
2	*Actinomadura madurae*	Am_7737_rv	5’-CTACACCCAGGACACCCCTA-3’
2	*Actinomadura madurae*	Am_7737_probe	5’-6FAM-TCGGCATATGAGAAAGCCTGGGA-BHQ1–3’
2	*Actinomadura pelletieri*	Ap_4241_fw	5’-CCTCTCGGATCTGCGTAG-3’
2	*Actinomadura pelletieri*	Ap_4241_rv	5’-GGTGCTGGTGCTGCA-3’
2	*Actinomadura pelletieri*	Ap_4241_probe	5’-CY5-AAGCCGAGACGTTCGAGATCCG-BHQ3-3’
3	*Streptomyces somaliensis*	Sso_8555_fw	5’-CGAGAAGTTCGTCAACGG-3’
3	*Streptomyces somaliensis*	Sso_8555_rv	5’-TAGCAGAGCCCGAGGAC-3’
3	*Streptomyces somaliensis*	Sso_8555_probe	5’ -TEXAS RED-CTGGCGCAGGCGATCAAGAA-BHQ2–3’
3	*Streptomyces sudanensis*	Ssu_22526_fw	5’-CGAAGCTACGGACCTTGAAC-3’
3	*Streptomyces sudanensis*	Ssu_22526_rv	5’-CTACTTCGAAGACCGCTTCC-3’
3	*Streptomyces sudanensis*	Ssu_22526_probe	5’-CY5-GTCTTCGAAGCGGCGGCCTT-BHQ3–3’

After design of the qPCR, the primers were validated against a smaller subset of DNA samples with classical PCR. For this a master mix was prepared consisting of 0.25 µL of forward primer (50 pmol/µl), 0.25 µL of reverse primer (50 pmol/µl), 10 µL of Roche FastStart PCR Master and 7.5 µl sterile deionized water. 18 µL of the mix was transferred to a PCR tube (Bio-Rad, Lunteren, The Netherlands) containing 2 µL of 2.5 ng/µl bacterial or fungal DNA. The PCR was run using a conventional PCR machine (Bio-Rad, Lunteren, The Netherlands) with a hot start at 94°C for 5 minutes, followed by 40 cycles of 94°C melting for 30s, 60°C annealing for 30s and 72 °C elongation for 1 minute. The amplicons were then visualized by gel electrophoresis on a 2.5% agarose gel (Sphaero Q, Gorinchem, The Netherlands). To assess if it is also possible to identify the causative agents to the species level with classical PCR, a quadruplex master mix was prepared consisting of 0.25 µL of forward primer Am_7737_fw (50 pmol/µl), 0.25 µL of forward primer Ap_4241_fw (50 pmol/µl), 0.25 µL of forward primer Sso_8555_fw (50 pM), 0.25 µL of forward primer Ssu_22526_fw (50 pM), 0.25 µL of reverse primer Am_7737_rv (50 pM), 0.25 µL of reverse primer Ap_4241_rv (50 pM), 0.25 µL of reverse primer Sso_8555_rv (50 pM), 0.25 µL of reverse primer Ssu_22526_rv (50 pM), 10 µL of Roche FastStart PCR Master and 6.0 µl sterile deionized water. 18 µL of the mix was transferred to a PCR tube (Bio-Rad, Lunteren, The Netherlands) containing 2 µL of 2.5 ng/µl bacterial or fungal DNA. The PCR was run using a conventional PCR machine (Bio-Rad, Lunteren, The Netherlands) with a hot start at 94°C for 5 minutes, followed by 40 cycles of 94°C melting for 30s, 60°C annealing for 30s and 72 °C elongation for 1 minute. The amplicons were then visualized by gel electrophoresis on a 2.5% agarose gel (Sphaero Q, Gorinchem, The Netherlands).

### Validation of singleplex qPCR

To assess the probe, a master mix was prepared consisting of 1 µL forward primer (50 pM), 1 µl reverse primer (50 pM), 1 ul probe (20 μM), 10 μL of LightCycler 480 Probes Master Mix and 5 µl DNA free water. Then 18 µL of master mix was transferred to a 96 well plate (LightCycler 480 multiwell, Roche, Basel) and mixed with 2 µL of DNA sample by resuspending three times. The 96-wells plate was sealed and placed in the LightCycler 480 (Roche, Basel, Switzerland) were it was run with an amplification program consisting of a 5 min preheating step at 95 °C, followed by 40 cycles consisting each of a 5s DNA denaturation step at 95 °C and a 30s annealing and extension step at 60 °C and ending with a 1 minute cooling step at 40 °C. The fluorescent output was measured at 465–510 nm, 533–580 nm, 595–615 nm and 618–660 nm and analyzed using the Abs Quant/Fit Points in the LightCycler 480 SW 1.5.1 program (Roche, Basel, Switzerland) with an analysis set to 35 cycles.

### Validation of multiplex qPCR

The multiplex qPCR was validated in three distinct steps. First, the probes were screened against a small subset of DNA samples in singleplex and multiplex to confirm the intended functionally of the probes. Second, the multiplex qPCR was screened against a larger DNA collection listed in Table 1. Third, the limit of detection (LOD) was determined based on a 10-fold dilution series ranging from 10 ng to 0.00001 ng of DNA isolated from *A. madurae* DSM43067*, A. pelletieri* DSM43383, *S. somaliensis* DSM40738 and *S. sudanensis* DSM43192. Here, the Quant-iT PicoGreen dsDNA Assay Kit (Invitrogen, USA) was used according to manufacturer’s instructions to accurately determine the DNA concentration of the DNA stocks. The LOD was determined for all three probes in triplicate in singleplex and multiplex. The data was analyzed as described above and the Cp values of each sample was plotted against the respective DNA concentration and visualized for each the respective probes. Finally, the qPCR was validated against a panel of clinical samples. These included 15 cultures and 14 grain samples (Table 2, S2 Table). ITS and 16S sequencing was used to identify the organism in the grain to species level. This validation was done using the LightCycler 480 (Roche, Basel, Switzerland) system as well as the Bio-Rad CFX96 Real-time system (Bio-Rad, Lunteren, The Netherlands). In the latter system each reaction consisted of 10 µL of Luna universal probe qPCR mix (New England Biolabs, Accra, Ghana), 0.8 µL of each primer, 0.4 µL of each probe, and RNase/DNase-free water to adjust the final volume.

### Statistical methods

To determine the sensitivity, specificity, positive predictive value and negative predictive value of the singleplex and multiplex qPCRs, the number of true- positive (TP), false- positive (FP), true-negative (TN) and false- negative (FN) test results were analysed with the chi- squared test (Graphpad).

## Results

### Genome sequencing

In total we sequenced 49 genomes and thereby expanded significantly the currently available genome sequences of key mycetoma agents *A. madurae* (13 extra genomes), *A. pelletieri* (13 extra genomes), *S. somaliensis* (1 extra genome) and *S. sudanensis* (13 extra genomes)*.*

**Fig 2:**
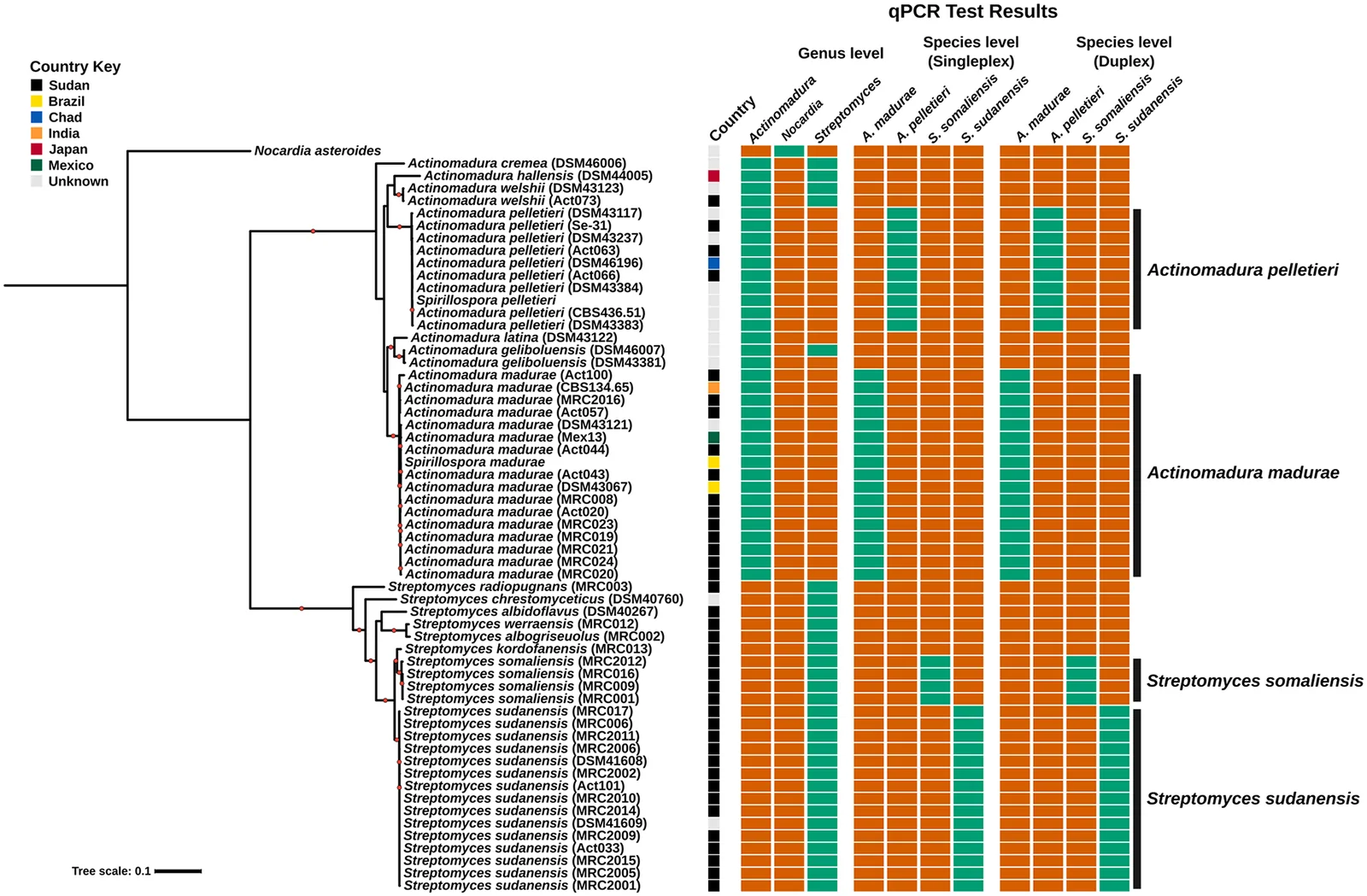
The phylogeny of actinomycetoma causative agents. Phylogenetic tree of conserved genes from the GTDB bac120 dataset, built de novo using iqtree2 with automatic model selection and other settings as described in the materials and methods. Red dots on branches represent ultrafast bootstrap support >95%. In this tree, the different tested isolates are depicted as well as their country of origin, and their positivity (green) or negativity (red) in the qPCRs designed.

Not unexpectedly, after sequencing the genomes it appeared that several of the isolates were previously misidentified and after comparing the whole genomes could now be assigned to a different species (S1 File). Our dataset included 8 new patient isolates of *A. madurae,* 3 isolates sourced from DSMZ and 2 from the CBS collection. The inclusion of these additional genomes revealed a new *A. madurae* population structure, with three distinct strongly supported sub-clades of *A. madurae* (Fig 2, S1 Fig, https://figshare.com/s/e2ce5c77aad51dd942ee). For the *A. pelletieri* isolates from the DSM and CBS culture collections, whole genome sequencing confirmed their identity.

The majority (n = 11) of the sequences originating from patients with a *Streptomyces* associated actinomycetoma were identified as *S. sudanensis.* This included 11 novel patient isolates from the Mycetoma Research Centre in Sudan and DSM41608 and DSM41609 previously deposited under the species *S. somaliensis* in the DSMZ collection, despite that previous molecular analyses already identified them as *S. sudanensis* [15]. Our whole genome-based approach supports this finding and suggests their annotation in DSMZ should be updated to reflect this molecular taxonomy. Only one new patient isolate from *S. somaliensis* was sequenced in this study, bringing the total number of available genomes to five.

### Identification of species-specific markers for qPCR

In total 38 species-specific gene families were identified for *A. madurae* and 61 for *A. pelletieri.* For *S. somaliensis* and *S. sudanensis,* a considerably lower number of species-specific gene families were identified, namely 1 for *S. somaliensis* and 7 for *S. sudanensis.* For *A. madurae, A. pelletieri* and *S. sudanensis,* initial tests of probes and primers designed against these gene families proved successful: unfortunately, for the single gene family identified for *S. somaliensis,* this failed. Therefore, new candidate gene-families were identified using relaxed criteria. For these relaxed criteria, we included gene families that were shared with close relatives, but where non-target species homologs shared a lower % sequence similarity to target sequences (<50%) (Table 4). This resulted in an additional 21 species-specific gene families for *S. somaliensis.*

**Table 4:** Identification of species-specific regions in the genomes of *Actinomadura madurae, Actinomadura pelletieri, Streptomyces somaliensis* and *Streptomyces sudanensis.*

Species	Species specific gene families per genome
*Actinomadura madurae*	38
*Actinomadura pelletieri*	61
*Streptomyces somaliensis*	1 (21)*
*Streptomyces sudanensis*	7

*Number of Species-specific gene families with relaxed criteria

### Development of *Actinomadura* multiplex amplicons to identify *Actinomadura madurae* and *Actinomadura pelletieri* to the species level

For *A. madurae* and *A. pelletieri,* there were 38 and 61 unique genomic regions identified, respectively. For the top candidates we developed primers based on the unique genome regions we identified. We developed primers and probes for selected unique regions. The primers were first tested with classical PCR to determine if these were species specific. When the primers were species specific qPCR probes were tested.

**Fig 3:**
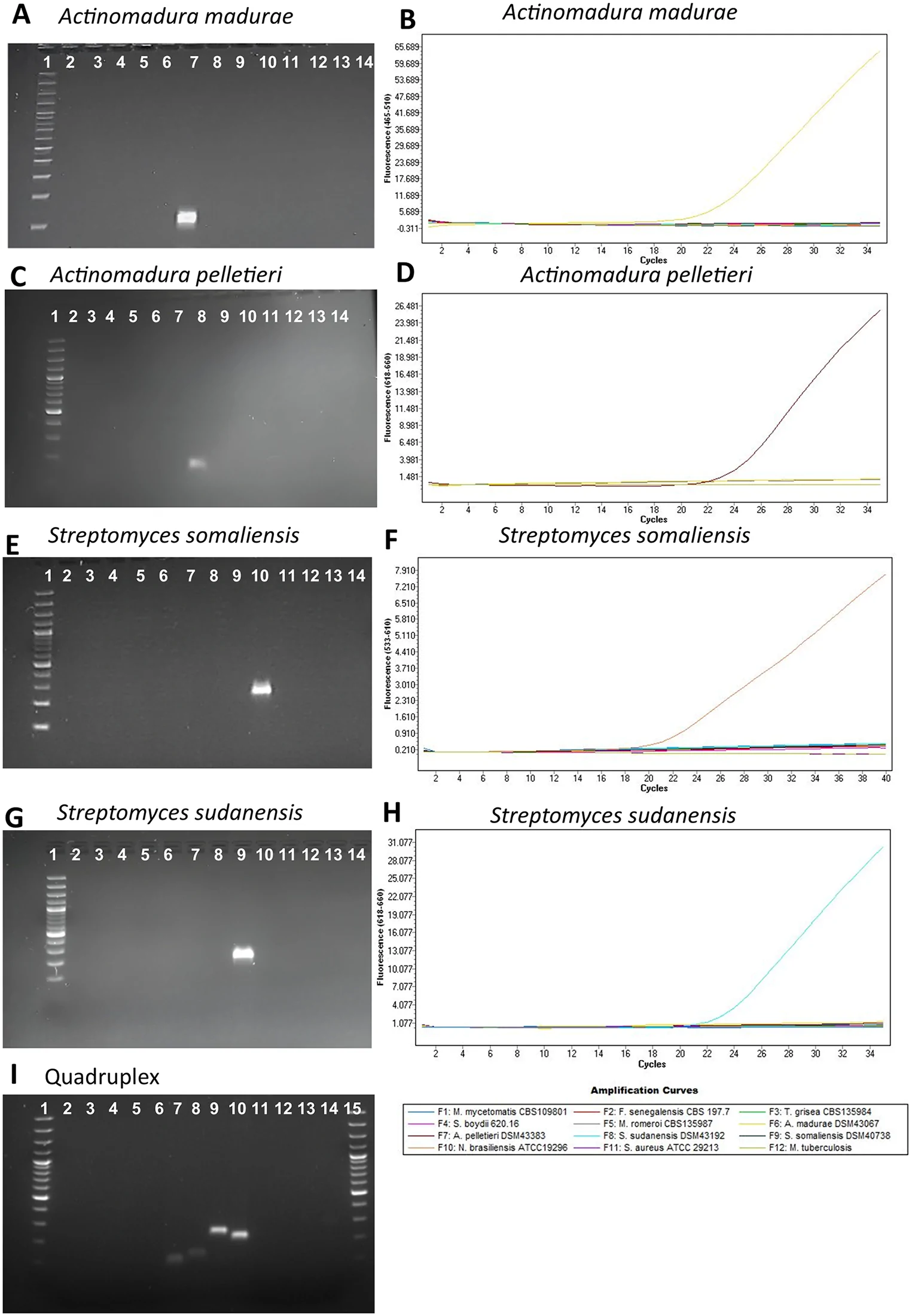
qPCRs developed for *A. madurae, A. pelletieri, S. somaliensis* and *S. sudaensis.* A: Gel electrophoresis demonstrating PCR products generated with the forward and reverse primers for *A. madurae*. **B:** qPCR with the *A. madurae* specific probe validated against 5 ng of DNA of DNA of 10 mycetoma causative agents, *S. aureus* and *M. tuberculosis*. The fluorescent signal was plotted against cycles. Fluorescent signal measured at 465–510 nm corresponding to the FAM fluorophore of the *A. madurae* probe. **C:** Gel electrophoresis demonstrating PCR products generated with the forward and reverse primers for *A. pelletieri*. **D:** qPCR with the *A. pelletieri* specific probe validated against 5 ng of DNA of DNA of 10 mycetoma causative agents, *S. aureus* and *M. tuberculosis*. The fluorescent signal was plotted against cycles. Fluorescent signal measured at 618–660 nm corresponding to the Cy5 fluorophore of the *A. pelletieri* probe. **E:** Gel electrophoresis demonstrating PCR products generated with the forward and reverse primers for *S. somaliensis* **F:** qPCR with the *S. somaliensis* specific probe validated against 5 ng of DNA of DNA of 10 mycetoma causative agents, *S. aureus* and *M. tuberculosis*. The fluorescent signal was plotted against cycles. Fluorescent signal measured at 595–615 nm corresponding to the TexasRed fluorophore of the *S. somaliensis* probe. **G:** Gel electrophoresis demonstrating PCR products generated with the forward and reverse primers for *S. sudanensis*. **H:** qPCR with the *S. sudanensis* specific probe validated against 5 ng of DNA of DNA of 10 mycetoma causative agents, *S. aureus* and *M. tuberculosis*. The fluorescent signal was plotted against cycles. Fluorescent signal measured at 618–660 nm corresponding to the Cy5 fluorophore of the *S. sudanensis* probe. I: quadruplex with classical PCR. For the electrophoresis pictures in panels **A, C, E, G and I,** the 100 bp ladder is depicted in lane 1. In lanes 2–10, the amplification products of the various mycetoma causative agents are shown. These are the amplification products of *M. mycetomatis* CBS109801 (lane 2), *F. senegalensis* CBS 197.79 (lane 3), *T. grisea* CBS135984 (lane 4), *S. boydii* CBS620.16 (lane 5), *M. romeroi* CBS135987 (lane 6), *A. madurae* DSM43067 (lane 7), *A. pelletieri* DSM43383 (lane 8), *S. somaliensis* DSM40738 (lane 9), *S. sudanensis* DSM41923 (lane 10), *N. brasiliensis* ATCC19296 (lane 11), *S. aureus* ATCC29213 (lane 12) and *Mycobacterium tuberculosis (*lane 13). The negative control is depicted in lane 14. The qPCRs in panels **B, D, F and H** were validated against 5 ng of DNA of *M. mycetomatis* CBS109801, *F. senegalensis* CBS 197.79, *T. grisea* CBS135984, *S. boydii* CBS620.16, *M. romeroi* CBS135987, *A. madurae* DSM43067, *A. pelletieri* DSM43383, *S. somaliensis* DSM40738, *S. sudanensis* DSM41923, *N. brasiliensis* ATCC19296, *S. aureus* ATCC29213 and *Mycobacterium tuberculosis*.

The *A. madurae* specific primers only amplified the DNA of *A. madurae* (Fig 3A), no amplification was noted for *A. pelletieri, N. brasiliensis, S. somaliensis, S. sudanensis* or the fungal species*.* The size of the amplicon was 102 base pairs, as expected. Likewise, the *A. pelletieri* specific primers only amplified the DNA of *A. pelletieri* (Fig 3C), the *S. somaliensis* specific primers only amplified the DNA of *S. somaliensis* (Fig 3E) and the *S. sudanensis* specific primers only amplified the DNA of *S. sudanensis* (Fig 3G)*.* No amplification was noted for the other mycetoma causative agents, *S. aureus* and *M. tuberculosis.* The size of the amplicon was 121 base pair for *A. pelletieri*, 204 base pair for *S. somaliensis* and 230 base pair for *S. sudanensis.* Due to the differences in amplicon size, it is possible to do species identification with classical PCR by using all four primers in one PCR reaction (Fig 3I).

Next, the species-specific probes were evaluated by qPCR. The number of cycles was set at 35, the time needed to complete these cycles was less than 1 hour. As shown in Fig 3B, only *A. madurae* had a positive signal with the *A. madurae* probe, none of the other bacterial species or the fungal species had a positive signal. Also the *A. pelletieri* probe (Fig 3D), the *S. somaliensis* probe (Fig 3F) and the *S. sudanensis* probe (Fig 3H) were species specific. They only gave a positive signal with their corresponding species.

**Fig 4:**
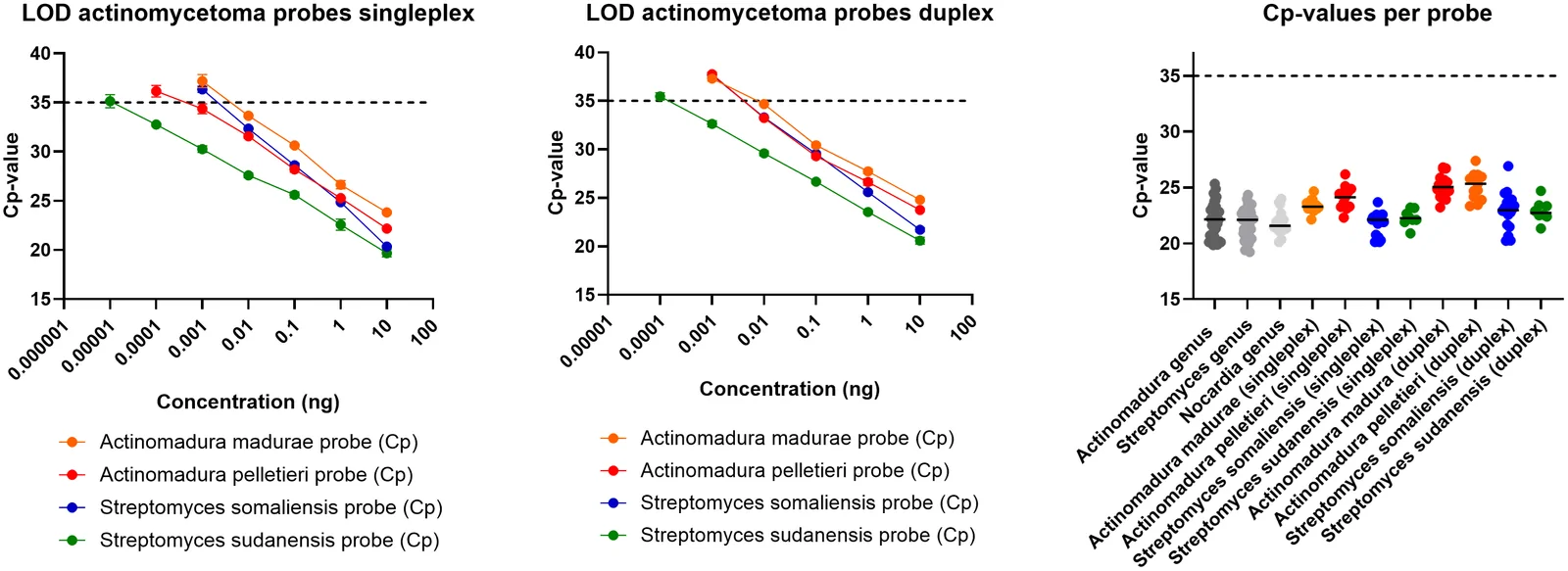
Limit of detection of the probes. A. Probes tested in singleplex against DNA derived from *A. madurae* DSM43067, *A. pelletieri* DSM43383, *S. somaliensis* DSM40738 and *S. sudanensis* DSM41923 B. Probes tested in duplex against DNA derived from *A. madurae* DSM43067, *A. pelletieri* DSM43383, *S. somaliensis* DSM40738 and *S. sudanensis* DSM41923 C. Cp-values of all *A. madurae*, *A. pelletieri*, *S. somaliensis* and *S. sudanensis* isolates tested using the *A. madurae, A. pelletieri, S. somaliensis* and *S. sudanensis* probes in singleplex or duplex.

When we tested the primers and probes against the entire strain collection (S1 Table), it appeared that none of the probes gave a positive signal against any of the 63 fungal DNA samples. The *A. madurae* probe gave a positive signal for 19 out of 19 *A. madurae* DNA samples, the *A. pelletieri* probe gave a positive signal for 13 out of 13 *A. pelletieri* samples, the *S. somaliensis* probe gave a positive signal for 7 out of 7 *S. somaliensis* samples and the *S. sudanensis* probe gave a positive signal for 19 out of 19 *S. sudanensis* samples. No cross reactivity was noted with the bacterial strains belonging to different genera. The mean cp-values for the *A. madurae, A. pelletieri, S. somaliensis* and *S. sudanensis* were 22.34 (21.22-23.23), 24.04 (range 22.31-26.17), 21.75 (range 20.12-23.68) and 22.30 (range 20.89-23.21), respectively (Fig 4C, Table 5 and S1 Table). This resulted in a 100% sensitivity and a 100% specificity for all probes **(Table 5)**.

**Table 5:** Limit of Detection (LOD), mean Cp-value and sensitivity and specificity of the singleplex and duplex assays.

	LOD (ng)	Mean Cp-value (range)	Sensitivity (%)	Specificity (%)
***A.madurae* probe**				
Singleplex on strains	0.00033	22.34 (21.22-23.23)	100	100
Duplex on strains	0.0018	23.62 (22.27-25.66)	100	100
Duplex on grains (ErasmusMC)	ND	26.02 (22.48-29.45)	100	100
Duplex on grains (University Saint-Berger)	ND	27.74 (26.17-29.74)	100	100
***A.pelletieri* probe**				
Singleplex on strains	0.00067	24.04 (22.31-26.17)	100	100
Duplex on strains	0.0014	24.67 (23.12-26.84)	100	100
Duplex on grains (ErasmusMC)	ND	25.58 (20.11-31.53)	100	100
Duplex on grains (University Saint-Berger)	ND	28.16 (21.30-32-13)	85.7	100
***S. somaliensis* probe**				
Singleplex	0.000017	21.75 (20.12-23.68)	100	100
Duplex	0.00015	22.91 (20.24-26.90)	100	100
***S. sudanensis* probe**				
Singleplex	0.0023	22.30 (20.89-23.21)	100	100
Duplex	0.0043	22.89 (21.33-24.68)	100	100

### The limit of detection of the qPCRs

Next, the limit of detection for each of the probes was determined. As can be seen in Fig 4, the LOD was 0.00033 ng bacterial DNA for the *A. madurae,* probe, 0.00067 ng for the *A. pelletieri* probe, 0.00056 ng for the *S. somaliensis* probe and 0.000013 ng for the *S. sudanensis* probe (Fig 4A, Tables 5 and S1).

### Multiplexing the probes

In the design depicted in Fig 1, the species-specific qPCRs will be run after the genus specific qPCR in a duplex fashion. This means that the *A. madurae* and *A. pelletieri* probes will be duplexed as well as the *S. somaliensis* and *S. sudanensis* probes. In the design, the dyes of the probes were chosen in such a way that they could also be multiplexed with the corresponding genus probe. As can be seen in Table 5 and Fig 4, the limit of detection of the individual species probes was higher when they were combined in the duplexes. The increase ranged from 1.87-fold for the *S. sudanensis* probe to 8.82-fold for the *S. somaliensis* probe. Despite this increase, all strains could be detected with the duplexes, indicating that running the qPCR duplex would not result in false negative results.

### Using the multiplex on clinical samples

To determine if the multiplex could be used on clinical samples we isolated DNA from 28 grain samples suspected for actinomycetoma or eumycetoma. As can be seen in Table 2, almost all *Actinomadura* grain samples were correctly identified, only one *A. pelletieri* grain sample was missed by the species-specific probe, but it was positive in the genus probe. This resulted in a sensitivity of 85.7% for the *A. pelletieri* probe when used in University Saint-Berger and a specificity of 100%. For the *A. madurae* probe a sensitivity and specificity of 100% was obtained in both institutes. Although the species-specific probes worked very well, we also noted that in 4 *Actinomadura* grain samples a positive reaction was also obtained for the fungal probe and that for 4 fungal grain samples also positive reactions were noted in the *Actinomadura* genus or *Streptomyces* genus probe. This indicates that the initial qPCR should always be followed by a species-specific qPCR to achieve a correct identification (S2 Table).

## Discussion

With the qPCRs we developed here, we were able to identify the causative agents in less than 2 hours. For the DNA extraction 30 minutes was needed and for the qPCR one hour. This is much faster than either histopathology, or culture, which take 2–15 and 7–14 days, respectively [12,37]. In the past, qPCR was often not used in mycetoma endemic regions because of the lack of real-time PCR machines. However, many qPCR machines were donated in Africa to aid with the COVID-19 diagnosis and these machines could be repurposed for diagnosis of mycetoma [38]. Furthermore, many manufacturers also worked towards miniaturizing qPCR machines to make them portable and field friendly. Examples include the MIC qPCR system used in the lab in a suitcase set-up for *Plasmodium falciparum* and *Plasmodium vivax* qPCRs [39] and the Biomeme Franklin mobile qPCR system used for the detection of Buruli Ulcer [40]. These developments show that qPCR can be done in field laboratories in relatively inaccessible parts of the world. In the current scheme we multiplexed the qPCR reactions in such a way that the maximum number of probes in a reaction is four, however, the scheme can be adapted depending on the endemic region, reducing the number of probes in a single reaction [41]. The most used qPCR machines can detect at least 3 different probes simultaneously. Furthermore, by designing the qPCR in an open system, it can be run using different machines and Taq polymerases. In our own study we validated the qPCR in two different machines, using two different polymerases and both worked well. However, for those labs which don’t have access to a qPCR of for whom the maintenance of the qPCR machines are too costly, we designed the qPCR in such a way that it can also be run with classical PCR. The first qPCR, discriminating actinomycetoma from eumycetoma was already designed in such a way to generate amplicons of different sizes which can be visualized by gel electrophoreses. Here we used the same approach. When all the primers are multiplexed in one classical PCR and the resulting products visualized by gel electrophoresis, *A. madurae, A. pelletieri, S. somaliensis* and *S. sudanensis* can be differentiated from each other due to the differences in the size of the PCR products (Fig 2I). Furthermore, we have also demonstrated that the qPCR could be successfully used on DNA isolated from actinomycetoma grains.

The qPCR scheme developed here is not only useful for diagnostic purposes. It could also be a useful tool for epidemiological surveys. In the past five years there have been 30 studies in which the epidemiology of mycetoma was studied in endemic regions [42–71]. The majority of these studies were performed in Africa. Only three studies used molecular identification for species identification: for the rest, species identification was based on histology and/or culture. The studies of Colom et al. [65], and Diongue et al. [70] both used barcoding sequencing, while that of Ahmed et al. [71] used a combination of a *M. mycetomatis* specific PCR and barcoding sequencing. Although these methods allowed the authors to also identify rare species to the species level, it is labour intensive and requires samples to be shipped for sequencing. That molecular identification is necessary to come to the correct species identification has been shown in several studies. In Sudan, for instance, it was demonstrated that from the 222 patients suspected to have mycetoma caused by *M. mycetomatis,* 10 were missed with histology and 16 were misidentified [12]. In the same subset of patients samples from 41 grains did not result in growth and 4 isolates were misidentified. In another study, also performed in Sudan, 142 of 991 mycetoma patients were diagnosed with actinomycetoma caused by *S. somaliensis* based on culture. From those 142, 133 were also identified by histopathology [13]. No *S. sudanensis* was identified. Since no molecular identification was used to confirm the identities, it could well be that several of the *S. somaliensis* cases were in fact *S. sudanensis.* Therefore, in order to make a proper estimation on the distribution of the causative agents world-wide, molecular identification should be included. The qPCR identification scheme developed here would make this possible, even in settings where barcoding sequencing is not available. At the moment, there is a prospective epidemiological survey performed by Drugs for Neglected Diseases initiative (DNDi) in India, Senegal and Ethiopia to determine the burden of mycetoma in these countries, including house-to-house visits. The described qPCR will be an excellent tool to determine the etiology of the causative agents in such surveys and will give more accurate data on the species distribution in these different regions.

The current qPCR scheme identifies the four most common causative agents of actinomycetoma in Africa, however, one of the shortcomings of our study was that this was based on data gathered in only two countries in Africa. Whether this etiology is similar in other African countries still needs to be determined. Therefore, this qPCR scheme should be validated in other actinomycetoma endemic regions, and based on the performance should be extended when other causative agents appear to be prominent in certain regions. For instance, when *A. welshii* appears to be one of the more prominent causative agents in a certain region an additional qPCR for that species could be added to the identification scheme. A second shortcoming is that, due to the limited number of patients tested with this qPCR scheme, it is difficult to assess the clinical impact on the patient of the qPCR system. To date, due to the long culture times, treatment is often started before the etiology is established. When implementing this qPCR scheme in different settings, the etiology will be established before treatment is started. It should therefore also be assessed what the impact will be on the clinical decision making and if this will have a direct outcome on the management of the patient.

## Supporting information

S1 File Whole genome sequencing and species identification. (DOCX)

S1 Table Isolates used in this study. In this table the isolates used, their source, identification, GenBank Accession numbers and qPCR results are found.
(XLSX)

S2 Table Grain samples used in this study. In this table the grains used, their source, identification, and qPCR results are found.
(XLSX)

S3 Table Characteristics of the sequenced genomes. (XLSX)

S1 Fig Phylogeny of *A. madurae.* A: Phylogenetic tree of complete genomes of *A. madurae. A. madurae* can be sepperated into three different clades. B. Average Nucleotide Identity Plot used in taxonomic assignment of *A. madurae* isolates. Pairwise average nucleotide identities were estimated using fastANI.
(DOCX)

S1 Raw Gel Raw images of gel electrophoresis pictures. (PDF)
