# Supplementary material for: A qPCR identification scheme to detect the most common causative agents of actinomycetoma in Africa
Source: PLoS Negl Trop Dis. 2026 Sep 8;20(9):e0014710. doi: 10.1371/journal.pntd.0014710 (PMC13577489; doi:10.1371/journal.pntd.0014710)
Supplement: Supplementary file 1 [file pntd.0014710.s006.pdf]

Actinomadura madurae

1 2 3 4 5 6 7 8 9 10 11 12 13 14 x x x

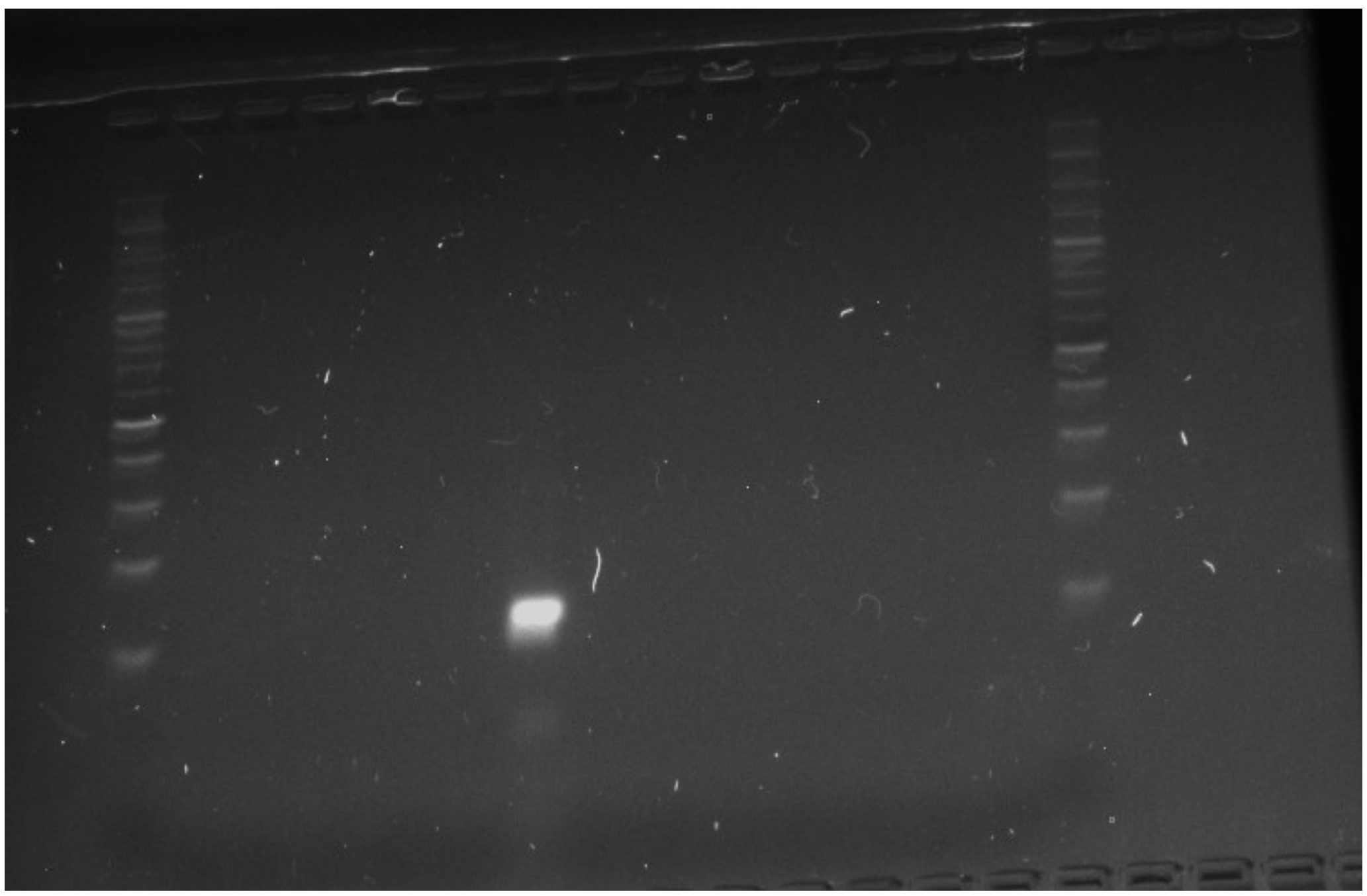

Gel electrophoresis demonstrating PCR products generated with the forward and reverse primers for *A. madurae*

the 100 bp ladder is depicted in lane 1. In lanes 2 to 10, the amplification products of the various mycetoma causative agents are shown. These are the amplification products of *M. mycetomatis* CBS109801 (lane 2), *F. senegalensis* CBS 197.79 (lane 3), *T. grisea* CBS135984 (lane 4), *S. boydii* CBS620.16 (lane 5), *M. romeroi* CBS135987 (lane 6), *A. madurae* DSM43067 (lane 7), *A. pelletieri* DSM43383 (lane 8), *S. somaliensis* DSM40738 (lane 9), *S. sudanensis* DSM41923 (lane 10), *N. brasiliensis* ATCC19296 (lane 11), *S. aureus* ATCC29213 (lane 12) and *Mycobacterium tuberculosis (*lane 13). The negative control is depicted in lane 14.

Actinomadura pelletieri

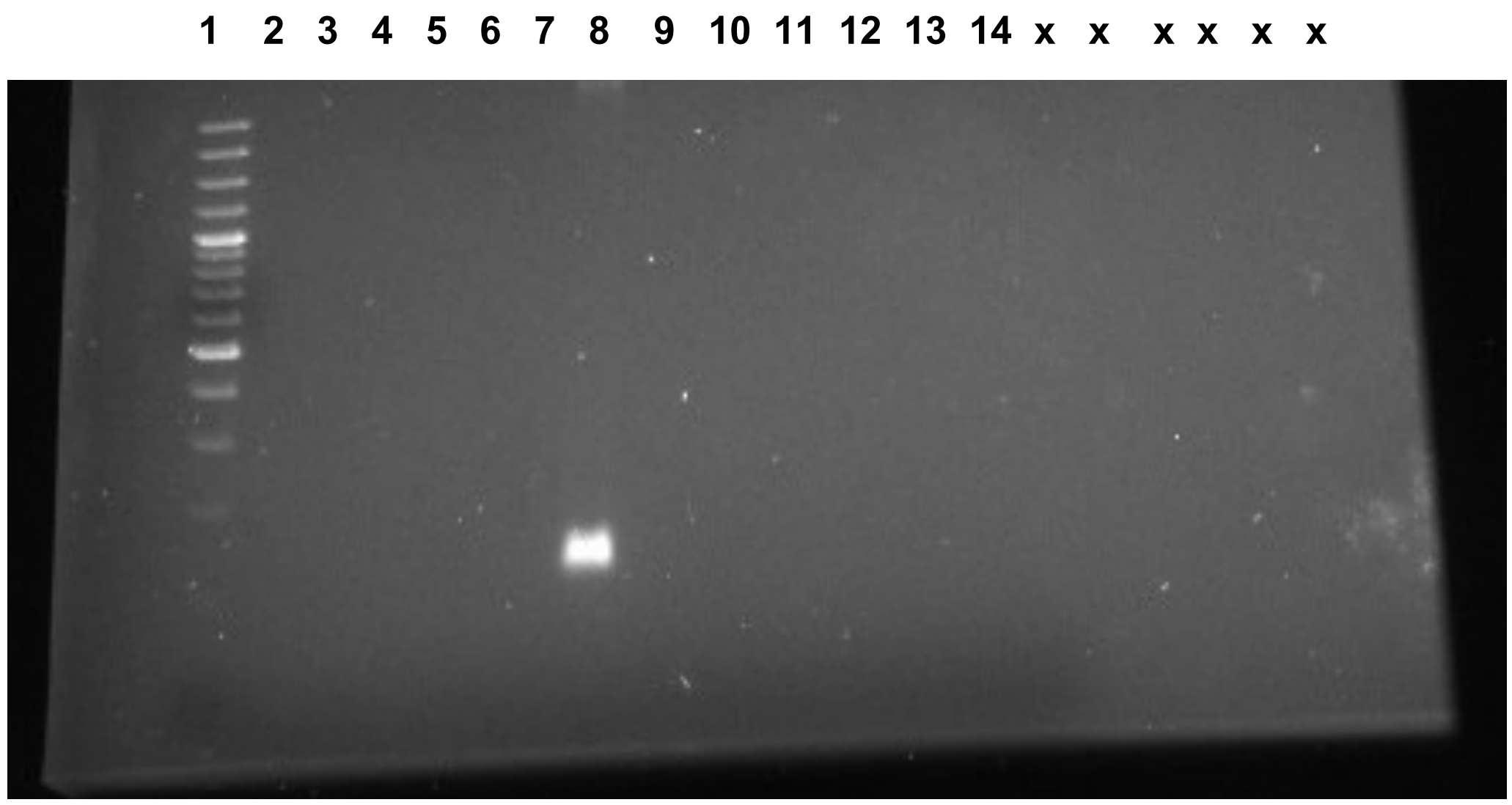

Gel electrophoresis demonstrating PCR products generated with the forward and reverse primers for *A. pelletieri*.

the 100 bp ladder is depicted in lane 1. In lanes 2 to 10, the amplification products of the various mycetoma causative agents are shown. These are the amplification products of *M. mycetomatis* CBS109801 (lane 2), *F. senegalensis* CBS 197.79 (lane 3), *T. grisea* CBS135984 (lane 4), *S. boydii* CBS620.16 (lane 5), *M. romeroi* CBS135987 (lane 6), *A. madurae* DSM43067 (lane 7), *A. pelletieri* DSM43383 (lane 8), *S. somaliensis* DSM40738 (lane 9), *S. sudanensis* DSM41923 (lane 10), *N. brasiliensis* ATCC19296 (lane 11), *S. aureus* ATCC29213 (lane 12) and *Mycobacterium tuberculosis (*lane 13). The negative control is depicted in lane 14.

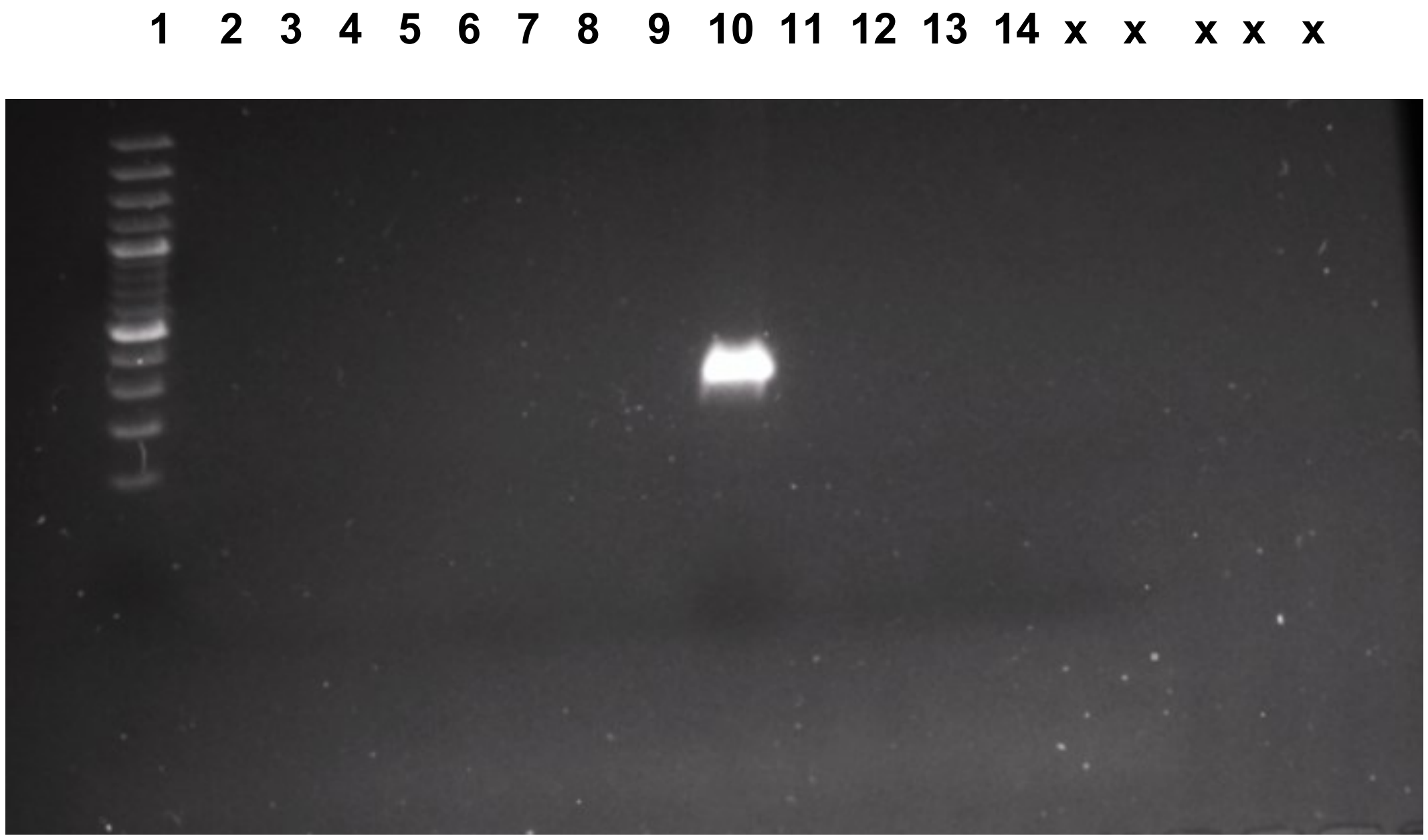

Gel electrophoresis demonstrating PCR products generated with the forward and reverse primers for *S. sudanensis*

the 100 bp ladder is depicted in lane 1. In lanes 2 to 10, the amplification products of the various mycetoma causative agents are shown. These are the amplification products of *M. mycetomatis* CBS109801 (lane 2), *F. senegalensis* CBS 197.79 (lane 3), *T. grisea* CBS135984 (lane 4), *S. boydii* CBS620.16 (lane 5), *M. romeroi* CBS135987 (lane 6), *A. madurae* DSM43067 (lane 7), *A. pelletieri* DSM43383 (lane 8), *S. somaliensis* DSM40738 (lane 9), *S. sudanensis* DSM41923 (lane 10), *N. brasiliensis* ATCC19296 (lane 11), *S. aureus* ATCC29213 (lane 12) and *Mycobacterium tuberculosis (*lane 13). The negative control is depicted in lane 14.

Streptomyces somaliensis

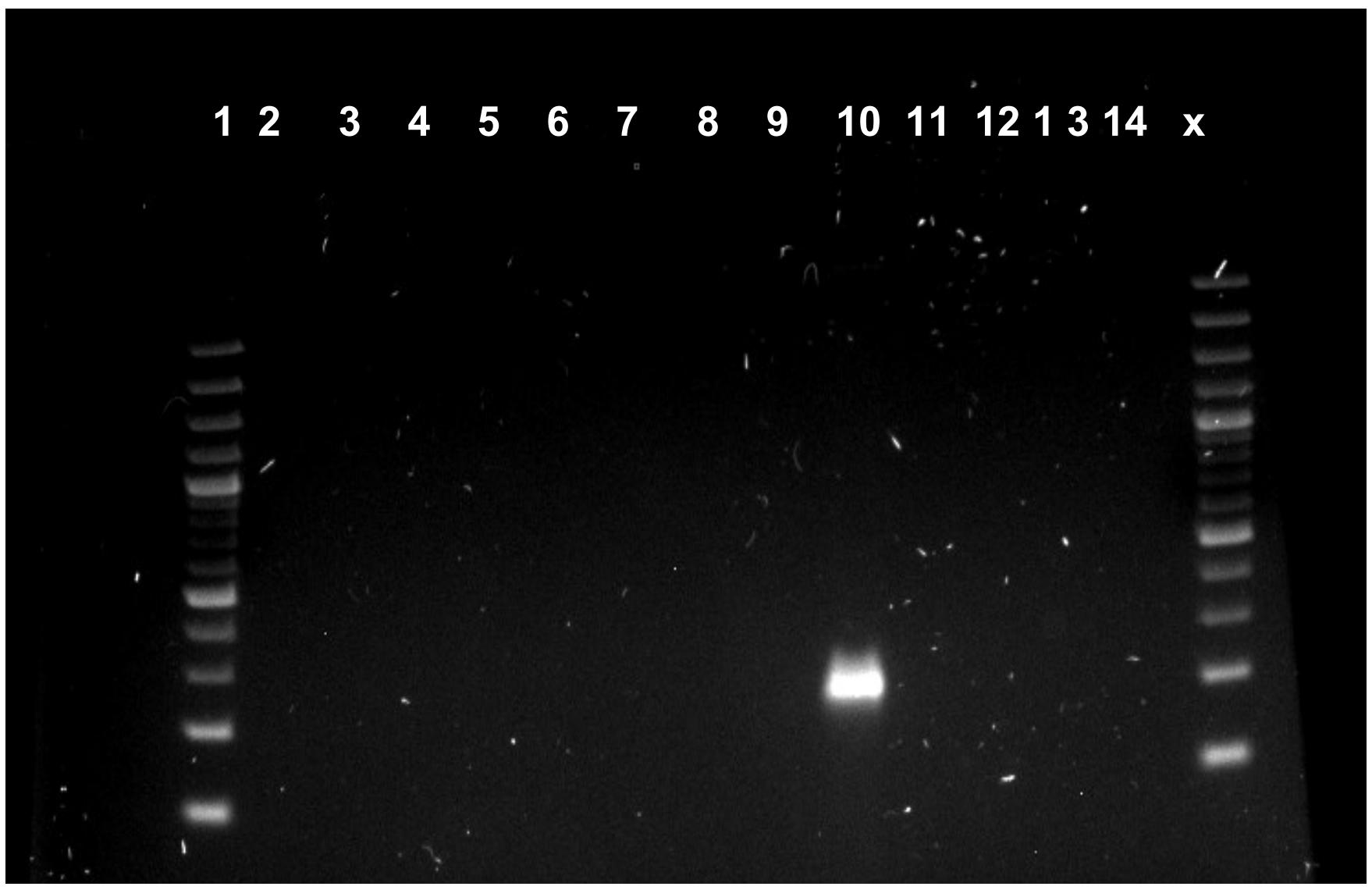

Gel electrophoresis demonstrating PCR products generated with the forward and reverse primers for *S. somaliensis*

the 100 bp ladder is depicted in lane 1. In lanes 2 to 10, the amplification products of the various mycetoma causative agents are shown. These are the amplification products of *M. mycetomatis* CBS109801 (lane 2), *F. senegalensis* CBS 197.79 (lane 3), *T. grisea* CBS135984 (lane 4), *S. boydii* CBS620.16 (lane 5), *M. romeroi* CBS135987 (lane 6), *A. madurae* DSM43067 (lane 7), *A. pelletieri* DSM43383 (lane 8), *S. somaliensis* DSM40738 (lane 9), *S. sudanensis* DSM41923 (lane 10), *N. brasiliensis* ATCC19296 (lane 11), *S. aureus* ATCC29213 (lane 12) and *Mycobacterium tuberculosis (*lane 13). The negative control is depicted in lane 14.

Quadruplex

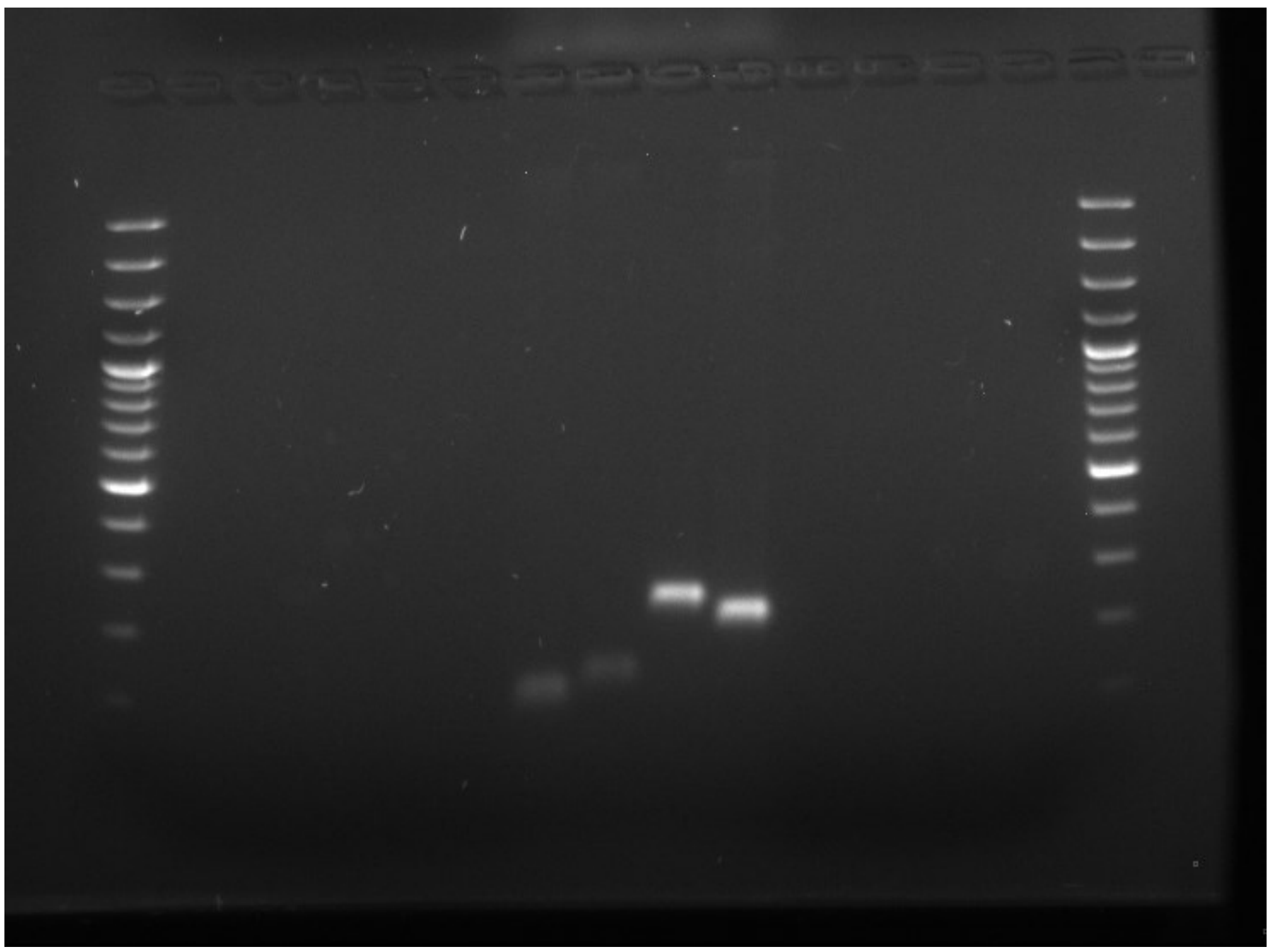

Gel electrophoresis demonstrating quadruplex classical PCR for the four bacterial species

the 100 bp ladder is depicted in lane 1. In lanes 2 to 10, the amplification products of the various mycetoma causative agents are shown. These are the amplification products of *M. mycetomatis* CBS109801 (lane 2), *F. senegalensis* CBS 197.79 (lane 3), *T. grisea* CBS135984 (lane 4), *S. boydii* CBS620.16 (lane 5), *M. romeroi* CBS135987 (lane 6), *A. madurae* DSM43067 (lane 7), *A. pelletieri* DSM43383 (lane 8), *S. somaliensis* DSM40738 (lane 9), *S. sudanensis* DSM41923 (lane 10), *N. brasiliensis* ATCC19296 (lane 11), *S. aureus* ATCC29213 (lane 12) and *Mycobacterium tuberculosis (*lane 13). The negative control is depicted in lane 14.
