# Supplementary material for: A qPCR identification scheme to detect the most common causative agents of actinomycetoma in Africa
Source: PLoS Negl Trop Dis. 2026 Sep 8;20(9):e0014710. doi: 10.1371/journal.pntd.0014710 (PMC13577489; doi:10.1371/journal.pntd.0014710)
Supplement: Supplementary file 2 [file pntd.0014710.s001.docx]

**Supplementary Information: Whole genome sequencing and species identification**

In total we sequenced 49 genomes and thereby expanded significantly the currently available genome sequences of key mycetoma agents *A. madurae* (13 extra genomes), *A. pelletieri* (13 extra genomes), *S. somaliensis* (1 extra genome) and *S. sudanensis* (13 extra genomes)*.*

Not unexpectedly, after sequencing the genomes it appeared that several of the isolates were previously misidentified and after comparing the whole genomes could now be assigned to a different species. From the isolates that were previously identified as *A. madurae,* whole genome sequencing identified DSM43381 and DSM46007 as *Actinomadura geliboluensis*, DSM44005 as *Actinomadura hallensis,* DSM46006 as *Actinomadura cremea,* and DSM46181 as a novel species of *Actinocorallia* with similarity to *Actinocorallia populi*, a species associated with plant roots. Except for DSM46007, of which the isolation source is unknown, all these isolates were either isolated from soil (DSM43381, DSM44005 and DSM46006)[33], mud from the Weida dam in Germany, or surface water from the Weida dam in Germany. We therefore propose the name *Nonomuraea weidayensis* for isolate DSM46182 and *Actinocorallia weidayensis* for isolate DSM46181. Since, these particular strains were originally isolated from the environment and were not linked with actinomycetoma before, we considered these as not associated with actinomycetoma. Strain DSM43123 was also originally identified as *A. madurae* but whole genome sequencing identified it as *Actinomadura welshii.* Also one or our patient isolates, ACT073 was identified as *A. welshii. A. welshii* was recently described as a new causative agent of actinomycetoma in Mexico [31] and ACT073 and DSM43123 seem to indicate that this *Actinomadura* species does cause mycetoma in Africa too. Our dataset included 8 new patient isolates of *A. madurae,* 3 isolates sourced from DSMZ and 2 from the CBS collection. The inclusion of these additional genomes revealed a new *A. madurae* population structure, with three distinct strongly supported sub-clades of *A. madurae* (**Figs 2, S1,** [**https://figshare.com/s/e2ce5c77aad51dd942ee**](https://eur01.safelinks.protection.outlook.com/?url=https%3A%2F%2Ffigshare.com%2Fs%2Fe2ce5c77aad51dd942ee&data=05%7C02%7Cw.vandesande%40erasmusmc.nl%7C7f90719cf09b48960e2c08debcbb963c%7C526638ba6af34b0fa532a1a511f4ac80%7C0%7C0%7C639155711031199479%7CUnknown%7CTWFpbGZsb3d8eyJFbXB0eU1hcGkiOnRydWUsIlYiOiIwLjAuMDAwMCIsIlAiOiJXaW4zMiIsIkFOIjoiTWFpbCIsIldUIjoyfQ%3D%3D%7C0%7C%7C%7C&sdata=U6KblD%2BtVjJ2F82MAyuEB4M98MRirTT2R%2BdxrM3UjIY%3D&reserved=0)**)**.  For the *A. pelletieri* isolates from the DSM and CBS culture collections, whole genome sequencing confirmed their identity.

The majority (n=11) of the sequences originating from patients with a *Streptomyces* associated actinomycetoma were identified as *S. sudanensis.* This included 11 novel patient isolates from the Mycetoma Research Centre in Sudan and DSM41608 and DSM41609 previously deposited under the species *S. somaliensis* in the DSMZ collection, despite that previous molecular analyses already identified them as *S. sudanensis* [13]. Our whole genome-based approach supports this finding and suggests their annotation in DSMZ should be updated to reflect this molecular taxonomy.  Only one new patient isolate from *S. somaliensis* was sequenced in this study, bringing the total number of available genomes to five. From the isolates which were previously identified as *S. somaliensis,* whole genome sequencing identified strain DSM41607, DSM41608 and DSM41609 as *S. sudanensis,* DSM40760 as *S. chresomyceticus* and DSM40267 and CBS947.68 as *Streptomyces albidoflavus. S. sudanensis* was already known as a mycetoma causative agent, especially in Sudan and again in this study we showed that in our cohort it is a common actinomycetoma causative agent. *S. albidoflavus,* however, has not yet been implicated as an actinomycetoma causative agent. DSM40267 and CBS947.68 are two copies of the same isolate which was originally isolated from a mycetoma patient and therefore might represent a new actinomycetoma species. However, without knowing how these strains were originally isolated it is impossible to draw strong conclusions. Within the patient isolates sequenced, the previously named *S. somaliensis* isolates MRC002, MRC003, MRC012 and MRC013 were *S. albogriseuolus, S. radiopugnanc, S. werraensis* and *S. kordofanensis.*
