# Supplementary material for: A qPCR identification scheme to detect the most common causative agents of actinomycetoma in Africa
Source: PLoS Negl Trop Dis. 2026 Sep 8;20(9):e0014710. doi: 10.1371/journal.pntd.0014710 (PMC13577489; doi:10.1371/journal.pntd.0014710)
Supplement: Supplementary file 3 [file pntd.0014710.s005.docx]

**S1 Fig.**

**S1 Fig. Phylogeny *A. madurae.* A:** Phylogenetic tree of complete genomes of *A. madurae. A. madurae* can be sepperated into three different clades. B. Average Nucleotide Identity Plot used in taxonomic assignment of *A. madurae* isolates. Pairwise average nucleotide identities were estimated using fastANI.
